# Robust microbe immune recognition in the intestinal mucosa

**DOI:** 10.1038/s41435-021-00131-x

**Published:** 2021-05-06

**Authors:** Olivier P. Schären, Siegfried Hapfelmeier

**Affiliations:** 1 Institute for Infectious Diseases, University of Bern, Bern, Switzerland; 2 Graduate School for Cellular and Biomedical Sciences, University of Bern, Bern, Switzerland

**Keywords:** Immunology, Population genetics, Antimicrobial responses

## Abstract

The mammalian mucosal immune system acts as a multitasking mediator between bodily function and a vast diversity of microbial colonists. Depending on host–microbial interaction type, mucosal immune responses have distinct functions. Immunity to pathogen infection functions to limit tissue damage, clear or contain primary infection, and prevent or lower the severity of a secondary infection by conferring specific long-term adaptive immunity. Responses to nonpathogenic commensal or mutualistic microbes instead function to tolerate continuous colonization. Mucosal innate immune and epithelial cells employ a limited repertoire of innate receptors to program the adaptive immune response accordingly. Pathogen versus nonpathogen immune discrimination appears to be very robust, as most individuals successfully maintain life-long mutualism with their nonpathogenic microbiota, while mounting immune defense to pathogenic microbe infection specifically. However, the process is imperfect, which can have immunopathological consequences, but may also be exploited medically. Normally innocuous intestinal commensals in some individuals may drive serious inflammatory autoimmunity, whereas harmless vaccines can be used to fool the immune system into mounting a protective anti-pathogen immune response. In this article, we review the current knowledge on mucosal intestinal bacterial immune recognition focusing on T_H17_ responses and identify commonalities between intestinal pathobiont and vaccine-induced T_H17_ responses.

## Ambivalent relationships between mucosal immunity and microbes

From the moment of birth, mammals experience countless transient or continuous exposures with a huge diversity of microbial species at their mucosal surfaces. The mucosal immune system responds to many, if not all of them. Depending on the type of microbe and its type of host interaction, microbe-induced mucosal immunity combines three main functions; firstly, to efficiently clear (or contain) and survive primary infection with a virulent pathogen; secondly, to prevent (or at least reducing severity of) secondary infection with the same or a similar pathogen; and thirdly, to tolerate the physiological colonization with beneficial and harmless microbes. As discussed further in the following sections, stereotypic pathogenicity-associated microbial behaviors, like epithelial cellular adhesion or host cell and tissue invasion have been linked to stereotypic types of immune responses [1]. For example, invasive bacteria tend to induce “type 1”, and epithelia adherent and tissue invading extracellular bacterial and fungal pathogens are typical inducers of inflammatory “type 3” immunity, associated with the induction of T_H1_ and T_H17_ helper T cells, respectively [2]. These are phenotypically distinguishable further from nonpathogenic microbiota-induced regulatory immunity, signified by the induction of peripherally induced regulatory T cells (pT_REG_) [3, 4], which are important to control inflammatory responses against indigenous nonpathogens that colonize the host continuously. All these and other so far conceptualized types of immune responses may generate long-lived antigen-specific circulating and tissue resident T-cell and B-cell populations. They also generate antibodies of antigen specificities and affinities and isotypes appropriate for the targeted microbe. The evolving concepts of immune response types and T-cell mediated intestinal mucosal immunity have been critically reviewed in detail elsewhere [4–6].

It is important to emphasize that the correct decision making by the mucosal immune system is crucial. Approximately 0.1% of the human population of the Northern hemisphere suffer from chronic inflammatory bowel diseases, manifesting as inflammatory immune responses against the nonpathogenic gut microbiota, which is believed to be the result of dysregulated antimicrobial mucosal adaptive immunity. The precise pathophysiologies of these diseases still remain enigmatic [7], and it is equally unclear how the other 99.9% of the population sharing the same environment and similar lifestyles successfully maintain host–microbial mutualism life-long.

## Innate immune recognition of microbial infection

How does the innate immune system discriminate between pathogens and nonpathogens to adequately and robustly activate and program the immune response according to the microbial threat? As first postulated in 1989 by Janeway [8], innate immunity employs germ-line encoded receptors (so-called pattern recognition receptors, PRRs) that inform the immune system of microbial presence or activity by sensing conserved microbe associated molecular patterns (so-called MAMPS) that are integral structural components or products of at least one type of microbe. Examples are lipopolysaccharides (LPS), flagellin, or viral nucleic acid molecules [9]. Toll-like receptors (TLR) and C-type lectin receptors (CLR) expressed on the cell surfaces of immune cells, epithelial cells, and other cell types respond to extracellular MAMPs [10, 11], whereas cytosolic PRRs like NOD-like receptors (NLRs) and RIG-I-like receptors (RLRs) scan the cytoplasm for the presence of MAMPs [12–15]. TLRs, CLRs, and NOD1 and NOD2 activation leads to transcriptional upregulation of pro-inflammatory cytokines (including pro-IL1β and pro-IL18), and/or Type I and Typ III interferons, depending on the combination of PRRs activated. The remaining NLRs respond to large variety of stimuli by activating the inflammasome leading to the proteolytic processing and release of IL1β and IL-18. In-depth reviews on PRR and inflammasome activation and signaling pathways can be found elsewhere [16–18]. It is important to note that most PRR agonists are conserved among deep phylogenetic groups of microorganisms and consequently cannot directly discriminate between pathogenic and nonpathogenic microbes. However, also pathogen-specific patterns are now known to, in principle, allow direct pathogen sensing through PRRs. For instance, bacterial type 3 secretion system needle proteins and cytosolic monomeric flagellin that tends to be injected by flagellated type 3 secretion system expressing pathogens into the host cell cytoplasm are both strong activators of the NAIP–NLRC4 inflammasome and subsequent pro-inflammatory IL-1 family cytokine and eicosanoid release [19–27]. The pyrin inflammasome, although not sensing any MAMP in the conventional sense, is activated by the common manipulations of host small Rho GTPases by various bacterial toxins [28]. The cytoplasmic muropeptide-specific PRR NOD1 has also been reported to be activated by excessive bacterial toxin mediated Rho GTPase activation [29].

Any microbial presence in body tissues that are normally sterile can be regarded as a good proxy for either infection by a barrier-breaching invasive pathogen or penetration of microbiota components due to barrier failure. PRR signaling in tissue macrophages and other cell types then activate an immune response locally at the site of microbial breach. Whenever microbes or their fragments become bloodborne, PRRs expressed on blood neutrophiles, platelets, and various other cell types throughout the body are activated to initiate a systemic immune response.

## Innate and adaptive immune maintenance of host–microbial mutualism at the mucosal border

This basic model of innate immune recognition requires several conceptual modifications to explain the distinct pro-immunogenic capacities of nonpathogens and pathogens as well as active immunosurveillance at the densely colonized mucosal barriers. In this review, we will focus on the intestinal mucosa, which is particularly well studied and constitutes the largest accumulation of microbes and mucosal immune cells in the body. First of all, the mucosal system is interfacing with a diverse and extremely dense microbial community producing enormous load of diverse MAMPs continuously, whereas pathogens may occur only transiently and at low relative abundancy. Several studies have suggested that TLRs are strategically expressed or signal only at the normally non-microbiota exposed basolateral cell surface of intestinal epithelial cells as an adaptation to this situation [30, 31], reviewed in [32]. New findings revealed that epithelial cellular TLRs are expressed also apically and able to sense luminal MAMP exposure, but are expressed in a complex anatomical pattern along the GI tract. TLR expression varies in quantity and subcellular localization depending on the gastrointestinal segment and age [33]. However, can this alone explain the discrimination between invading pathogens and nonpathogenic microbial cells (or microbial fragments) spontanously translocating through minor epithelial defects commonly induced by dietary and pharmacological stressors? The intestinal mucosal barrier is heavily guarded with tissue resident innate immune cells, including an enormous density of intestinal macrophages. The main population of macrophages in the resting colon has been characterized (in mice) as Ly6C^−^ CD11b^+^, MHCII^+^, CX3CR1^hi^ F4/80^+^ CD64^+^ CD11c^+^ cells [34, 35]. Ly6C^high^ blood circulating monocytes continuously renew this macrophage population by full differentiation into a non-inflammatory phenotype (also referred to as “regulatory” or “microbe anergic” macrophages). They function as efficient scavengers of spontaneously translocating (usually harmless) microbes but are hyporesponsive to pro-inflammatory stimuli including TLR agonists [36, 37]. They can be seen as a phagocytic buffer eliminating low-grade microbial contamination in the mucosal lamina propria and thereby define an immunological setpoint below which no inflammatory response is triggered. These macrophage phenotypes maintain a local anti-inflammatory cytokine milieu indirectly by efficiently eliminating microbes, as well as directly by producing anti-inflammatory cytokines like IL-10. This maintains a local state of innate immune hyporesponsiveness to maintain tissue integrity.

Local immune homeostasis is stabilized further by an active process of adaptive immunosurveillance of non-infection-associated microbial antigen. Owing to the low permeability of the mucus and epithelial barrier for nonpathogenic microbes, combined with the aforementioned scavenging of residual microbial tissue contaminants by resident macrophages, the homeostatic immune presentation of nonpathogenic microbes depends on active immune sampling of microbes from the luminal mucosal surface. This occurs mainly through specialized epithelial microfold cells (M cells) in the follicle associated epithelia of Peyer’s patches and the smaller isolated lymphoid follicles that transcytose luminal microbes to underlying antigen presenting dendritic cells [38]. This homeostatic immunosurveillance drives the continuous induction of nonpathogenic microbe-binding T cell-dependent and -independent intestinal secretory IgA [39] and microbe-specific peripherally induced regulatory T cells (pT_REG_) expressing RORγt and FoxP3. pT_REG_ home back to the intestinal mucosa where they inhibit the induction of inflammatory immune responses and consequently stabilize host–microbial mutualism [3, 40–42] in an antigen-specific manner [43]. pT_REG_ differentiation is augmented by dietary and microbial metabolites, such as dietary retinoic acid (Vitamin A) and butyrate [44, 40, 45]. Butyrate is one of the main fermentation end products accumulated by an intact anaerobic colonic microbial community and the main carbon source for colonic enterocytes in adults, and thus signifies intact host–microbial mutualism. Whether among a complex microbiota, discrete species of microorganisms are stronger inducers of pT_REG_ than others as suggested by gnotobiotic mouse experiments still remains unclear [41, 44]. This mucosal adaptive immune education by indigenous gut microbes is important to stabilize mutualism also during episodes of intestinal epithelial attrition from dietary, pharmacological or pathogenic microbial insults. Epithelial barrier damage tends to induce pathogenic T_H1_ or T_H17_ responses against indigenous bystanders, which would destabilize host–microbiota mutualism [46]. In such situations previously induced pT_REG_ cells have been reported to expand rapidly in response to cognate microbial antigen to avoid the de novo induction of pro-inflammatory anti-commensal T-cell responses [47].

Infection with a virulent mucosal pathogen, however, can (or needs to) temporarily break down host–microbial mutualism. Extensive tissue injury and/or intracellular or interstitial microbial infection of host tissue shift the cytokine environment to a pro-inflammatory state, driven by the release of pro-inflammatory mediators such as IL-1β, IL-18, IL-6, and TNFα by various cell types including epithelial cells and innate lymphoid cells. In the early phase of acute inflammation, activated endothelia and released chemokines drive the influx of mainly neutrophils from the circulation that become activated by pathogen contact directly or by cytokines to release bactericidal substances that kill and contain microbes. In case of a virulent pathogen, the acute inflammation may not be sufficient to clear the infection, in which case the inflammatory exudate subsequently becomes more dominated by recruited blood monocytes, which in the inflammatory cytokine microenvironment differentiate into inflammatory macrophages [34], as well as T cells. The inflammatory T-cell infiltrate is dominated by large numbers of cytokine producing effector T cells. These have been shown in many bacterial infection models to be critically important for clearing primary infection (including in *Citrobacter rodentium* [48] and non-typhoidal *Salmonella enterica* infection [49] infection models).

IL-17 and IL-22 producing so-called “T_H17_” cells are CD4^+^ T cells predominantly induced by extracellular epithelial adherent intestinal pathogens. One of best-studied examples is the mouse pathogen *Citrobacter rodentium*, a surrogate mouse model for human EHEC and EPEC infection [48]. T_H17_ cells are important to curb early *C. rodentium* infection by strengthening epithelial barrier function through IL-22 signaling [50]. Mucosal pathogens that invade the mucosa through intracellular infection, such as *Salmonella enterica*, additionally induce IFNγ-producing CD4 effector cells, referred to as T_H1_ cells, and CD8 effector T cells (reviewed in [6, 51]) that are also recruited into the infected mucosal tissue. These components of the T-cell response are important to control intracellular infection, although the precise contributions of different T-cell subsets in *Salmonella* infection remain unclear due to the diversity of mouse infection models used in the field.

Effector T-cell populations can become highly enriched in the inflamed infected mucosal tissue and give rise to recirculating effector memory cells (T_EM_) and non-recirculating tissue resident memory cells (T_RM_) that may persist long-term following resolution of primary infection to protect against secondary infection. The work by many researchers using various infection models provides ample evidence that, like innate immune defense, the inflammatory effector T-cell mediated defense against a virulent mucosal pathogen is generally PRR-signaling dependent [52].

## Homeostatic pathobiont-induced immune responses

T_H17_ T-cell responses have been studied extensively not only owing to their key role in promoting mucosal inflammation and clearing primary infection with a virulent mucosal pathogen. Particular research attention is also focused on the causal role of intestinal induced T_H17_ cells as drivers of autoimmune pathologies such as shown in experimental autoimmune encephalomyelitis (EAE) in mice [53], a model for multiple sclerosis in humans. The T_H17_ T cells responsible are commonly referred to as “pathogenic” T_H17_ cells. However, T_H17_ cells have originally been described in the context of a physiological immune response to certain commensal intestinal bacteria, the best-defined of which is Segmented Filamentous Bacterium (SFB; *Candidatus Arthromitus* sp. [54]). SFB has long been known as a dominant inducer of physiological microbiota-induced intestinal IgA in rodents [55], which is not associated with mucosal inflammation and pathology, despite the characteristic intimate adherence of SFB to intestinal epithelial cells that is reminiscent of adherent bacterial pathogens. SFB has been found to be the main inducer of baseline levels of T_H17_ cells found in most commercially available conventional mouse strains, while germ-free mice have few if any T_H17_ cells in the intestinal lamina propria [56]. SFB is a paradigm for microorganisms commonly found in healthy hosts referred to as “pathobionts”. The term pathobiont attempts to categorize symbiotic microorganisms whose mutualistic relationship with the host depends on a functioning immune system and consequently can cause tissue- or immunopathology in immunodeficient hosts [57]. Despite their pathogenic potential, they normally appear to play a mostly beneficial immunostimulatory role. For example, murine SFB colonization-associated homeostatic intestinal T_H17_ cells have been demonstrated to confer a certain degree of “cross-protectivity” against the unrelated T_H17_-inducing pathogen *C. rodentium* [56], as they produce epithelial-protective cytokines. Although the relevance of SFB or similar species in humans is not clear [58], also healthy human subjects have been demonstrated to commonly harbor intestinal T_H17_ inducing bacterial species [1].

**Fig. 1:**
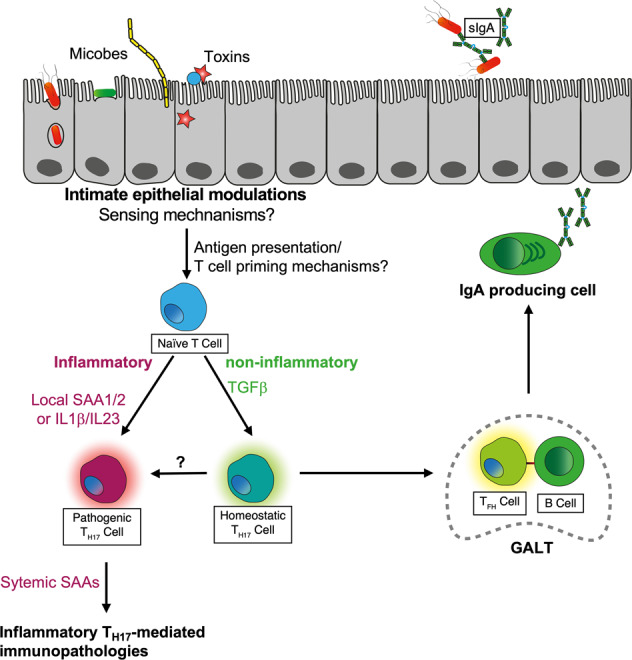
Induction of T_H17_ cells with divergent phenotypes by epithelium modulating microbes and microbial toxins. Intimate epithelial modulations by microbes and microbial toxins drive the differentiation of distinct T_H17_ phenotypes driving systemic immunopathology (“pathogenic T_H17_ cells) or trans-differentiating into follicular helper T (T_FH_) cells in gut-associated lymphoid tissues (GALT). sIgA, secretory IgA; SAA, serum amyloid protein.

It is now established that “homeostatic” T_H17_ cells, such as those induced by SFB in immunocompetent hosts, and virulent infection-induced “pathogenic” T_H17_ cells are phenotypically distinct. The former have a very low, the latter a high propensity to participate in inflammatory pathology and to disseminate systemically [59]. Recent work by Lee and coworkers identified serum amyloid A proteins (SAAs) as key promoters of the differentiation of “pathogenic” T_H17_ cells. Local epithelial produced SAA1 and 2 was shown to amplify cytokine production by SFB-induced T_H17_ cells substituting both TGFβ and IL-23, although the initial T-cell priming mechanism remains unknown. Systemic SSAs, known markers of generalized inflammatory disease, were identified as key mediators of “pathogenic” T_H17_ cell-mediated systemic immunopathologies [60]. T_H17_ cells demonstrate a high degree of plasticity and were recently shown to transdifferentiate into follicular helper T cells (T_FH_), which is essential for T-dependent intestinal IgA responses [61]. Figure 1 summarizes the emerging concept. Recent work has further established that in absence of inflammation, T_H17_ can transdifferentiate also into pT_REG_ cells [62] and that all microbiota-induced pT_REG_, which like T_H17_ cells express RORγt, apparently are derived from T_H17_ cells [3]. The reported trans-differentiation of also gut microbe-induced pT_REG_ into intestinal T_FH_ and their role in intestinal IgA induction remains controversial [61, 63]. Also, to which extent homeostatic T_H17_ responses have an immune correlate in “homeostatic” pathobiont-induced mucosal T_H1_ T cells, as recent work indicated [64], or mucosal CD8 T cells is currently not well understood.

## Commonalities between mucosal pathobiont- and vaccine-induced immunity

Collectively, all this information suggests that the efficacious induction of protective high-affinity IgA by mucosal vaccines may also depend on the capacity to primarily induce T_H17_ cells (see Fig. 1). Hence, there may be important lessons to learn from intestinal pathobionts regarding the design of mucosal vaccines.

Firstly, stereotypic intimate microbial-epithelial interactions appear to signify the induction of T_H17_ and consequent T-dependent IgA responses. Thus, rat isolates of SFB that are epithelial attachment-deficient in mice, like epithelial attachment/effacement-deficient mutants of *C. rodentium*, are unable to induce protective T_H17_ responses [1]. Epithelial adhesion may be a general microbial behavioral pattern driving T_H17_ responses [1], but also other intimate microbial-epithelial interactions may drive T_H17_ and high-affinity IgA. Cholera toxin (CT), a proven IgA biasing mucosal adjuvant in mice, is a potent T_H17_ inducer [65, 66]. Moreover, CT depends on T_H17_ cells to induce CT-specific intestinal IgA [61]. The toxicity of wild-type CT makes it unsuitable for direct human medical application as adjuvant. However, also nontoxic CT variants that have been developed for this purpose and have been shown to share this T_H17_-polarizing effect, which depends on their residual toxicity [66]. Work carried out in our laboratory [67] systematically addressed the specific effect of bacterial epithelial invasiveness (rather than adhesiveness) in the microbial induction of protective mucosal immunity in non-typhoidal invasive salmonellosis. A transitory mucosal bacterial colonization model in germ-free mice [68] was applied to carry out mucosal immunizations with invasive and noninvasive versions of an non-replicative auxotrophic strain of *Salmonella enterica* serovar Typhimurium (*S*. Typhimurium) [67]. Being unable to proliferate inside host cells and tissues, invasive auxotrophic *S*. Typhimurium was found to be a virulent but capable of transient epithelial-restricted cell invasion [67]. The high-affinity IgA-mediated [69] efficacy of auxotrophic *S*. Typhimurium strongly depended on this epithelium-restricted invasiveness, as immunization with live but noninvasive mutants was poorly protective, comparable to chemically inactivated bacteria. T_H17_ and other effector T-cell subsets were, however, not directly investigated.

A second emerging pattern is the remarkable redundancy of PRR signaling demonstrated in pathobiont, as well as live mucosal vaccine, -induced immunity. SFB in mice was found to induce normal numbers of T_H17_ cells also in knockout animals lacking all TLR- and IL1R-family-signaling (MYD88/TRIF double-deficient) or NOD1/NOD2-signaling [56, 70]. Earlier seminal work by Gavin and coworkers demonstrated that also adjuvant-dependent serum antibody responses to model antigens can be robustly induced in MYD88-/TRIF-deficient mice [71], first raising the hypothesis that the main mammalian PRRs are essential for innate defense against pathogens, but potentially redundant for the induction of adaptive immunity. This hypothesis has since been supported by the demonstration of extensive cooperative flexibility between innate and adaptive immunity in protection against mucosal opportunistic infections, involving CD4 T cell-dependent antibody immunity [72]. In our own work using auxotrophic *S*. Typhimurium (see also previous section) we made similar observations in live vaccine-induced immunity. In absence of inflammation, major PRR-signaling pathways through MYD88 & TRIF (all TLR and IL1R family signaling), NOD1 and NOD2, Caspase1/11 (canonical and noncanonical inflammasome activation), and NLRC4 were individually redundant for protective immune induction, despite a demonstrated role in (inflammatory) innate immune defense against virulent *S*. Typhimurium infection [73–75].

## Final conclusion and perspective

Powerful human live attenuated vaccines, such as the yellow fever vaccine, very likely signal through several different PRRs, including TLRs, although the detailed PRR requirements for vaccine efficacy remain understudied [76]. The approach to increase immunogenicity of non-live vaccine by combination with potent TLR- or inflammasome activating adjuvant substances is certainly a proven strategy for parenteral immunization. However, effective mucosal immunity protective against mucosal infection is most adequately induced through the mucosal route. The development of novel mucosal adjuvants has been formulated as a priority research area in vaccinology and in mucosal immunology. However, the work reviewed above highlights the importance of a second priority area, the development of more effective vaccine delivery systems, as a promising future direction. Pathobionts, pathogens, and bacterial toxins trigger key inductive steps in the induction of protective mucosal immunity that, importantly, appear to be systematically decouplable from the adverse inflammatory processes that drive immunopathology. However, the idea to optimize mucosal vaccines to be potent inducers of T_H17_ has the important caveat that we still lack a clear understanding of which factors can deviate homeostatic T_H17_ responses towards the generation of pathogenic T_H17_ cells that could drive autoimmune pathologies. At the current pace, future research in mucosal immunology and vaccinology certainly holds promise to close this and other remaining knowledge gaps.
